# Effects of Cardiac Contractility Modulation on Right Ventricular and Left Atrial Strain in Patients with Chronic Heart Failure

**DOI:** 10.3390/jcm14134484

**Published:** 2025-06-24

**Authors:** Cornelia Raab, Peter Roehl, Matthias Wiora, Henning Ebelt

**Affiliations:** Department of Medicine II, Catholic Hospital “St. Johann Nepomuk”, Haarbergstr. 72, 99097 Erfurt, Germany

**Keywords:** cardiac contractility modulation, RV strain, LA strain, heart failure

## Abstract

**Background:** Cardiac contractility modulation (CCM) is an established therapy for patients with heart failure with a reduced ejection fraction (HFrEF) who are still symptomatic despite guideline-directed medical therapy. It has been described previously that CCM leads to both an improvement of heart failure symptoms as well as of the parameters of left ventricular (LV) function, including LVEF and global longitudinal strain (GLS). However, so far there are no reports describing the effects of CCM on right ventricular (RV) or left atrial (LA) function, respectively. This might be of particular interest as RV global strain (RV GS) and LA strain are important prognostic parameters in heart failure. **Methods:** Adult patients with heart failure with reduced left ventricular function (LVEF <45%) and a QRS complex <130 ms despite guideline-directed medical therapy and with an indication for CCM were eligible for inclusion into this study. Patients receive a follow-up examination every 3 months, including a standardized echocardiographic examination with a special focus on strain analysis. While the effects of CCM on LV global longitudinal strain have been described before, this analysis reports the findings on the RV and LA strain. **Results:** Between 30.12.2021 and 10.09.2024, 22 patients were prospectively included in the study. CCM implantation was performed in 19 patients. Under active CCM therapy, there was an improvement in right ventricular global strain (CCM: −13.7 ± 4.5 vs. no CCM: −10.1 ± 5.0; *p* < 0.05), free wall strain (CCM: −14.6 ± 7.3 vs. no CCM: −10.3 ± 10.2; *p* < 0.05), left atrium strain rate (CCM: 19.7 ± 1.0 vs. no CCM: 15.3 ± 10.2; *p* < 0.05), and left atrium strain contraction (CCM: −11.5 ± 7.0 vs. no CCM: −7.1 ± 8.5; *p* < 0.05), whereas there was no difference in left atrium strain conduit (CCM: −9.0 ± 5.0 vs. no CCM: −8.1 ± 5.4; n.s.). To determine which of these parameters are linked to an improvement of quality of life, as seen in the Kansas City Heart Failure Questionnaire (KCCQ), a regression analysis was performed. It turned out that only the parameters of left atrial (LA) strain (LAS_R and LAS_CT) were significantly associated with improved quality of life, while other echocardiographic parameters, such as LV-EF, LV-GLS, and RV-GS, showed no clear association. **Conclusions:** CCM therapy is not only associated with improvements of left ventricular function but also restores right ventricular and left atrial strain in patients with HFrEF. Regarding the improvement in quality of life, the increase of LA strain seems to be of special importance.

## 1. Background

Speckle tracking echocardiography (STE) is a non-invasive method for the quantitative assessment of left ventricular myocardial function [1]. It enables an objective, user-independent analysis of myocardial deformation during the entire cardiac cycle. Strain analysis of myocardial deformation is based on measuring the change in length of a myocardial segment relative to the end-diastolic length: strain (%) = [length_enddiastolic_ − length_endsystolic_)/length_enddistolic_] × 100. A distinction is made between longitudinal strain (shortening along the longitudinal axes, negative values), circumferential strain (shortening in the cross-section, negative values) and radial strain (wall thickening, positive values). STE (speckle tracking echocardiography) uses reflection patterns (“speckles”) in the myocardial tissue, the movement of which is tracked by dedicated software. This allows the regional and global myocardial function to be precisely assessed.

To quantify right ventricular (RV) function during transthoracic echocardiography, the “Tricuspid Annular Plane Systolic Excursion” (TAPSE, linear measurement in mm of how far the tricuspid valve annulus moves upwards during systole) is commonly used. However, there is also the possibility of speckle tracking analysis of the RV [2]. For the determination of right ventricular longitudinal strain, only one slice plane is used, which is shown in a 4-chamber view focusing on the right ventricle. It is important that the RV apex, the free wall of the RV, and the interventricular septum are all clearly visible. During the analysis, either only the free right ventricular wall is assessed (right ventricular free wall strain, RV FWS) or all segments are assessed to determine global right ventricular strain (RV GS), respectively [3]. Reference values have already been published for both parameters, although gender-specific differences should be considered. The mean RV GS in healthy volunteers has been reported as −25.8 ± 3.0% and the mean RV free wall strain has been reported as −30.5 ± 3.9%, whereas RV FWS under −23% and RV GS under −20% are considered pathological [3,4]. The prognostic importance of RV GS has been established in the setting of pulmonary hypertension, arrhythmogenic right ventricular cardiomyopathy, or right myocardial infarction, respectively [5,6]. Several studies have also demonstrated the prognostic significance of RV GS for overall survival and hospitalization rates. In a large comparative study of right ventricular assessment with cardiac MRI, RV strain based on speckle tracking was shown to be a very strong and independent predictor of all-cause and cardiovascular mortality in HFrEF patients [7].

**Figure 1:**
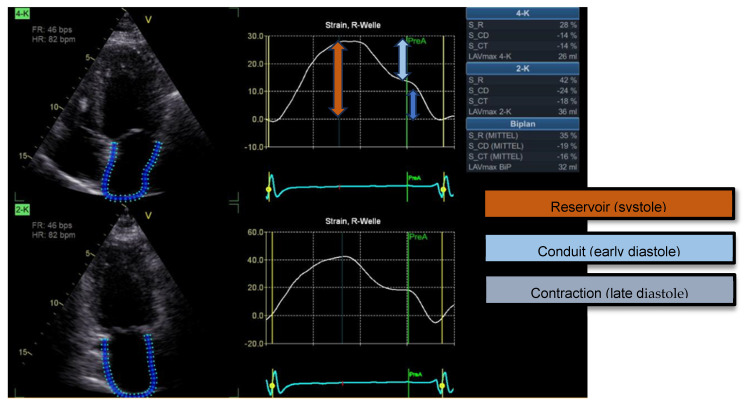
Example of left atrial strain analysis using speckle tracking echocardiography.

Another important parameter in acute and chronic heart failure is the assessment of LA function. LA size is typically measured planimetrically using the standardized four- and two-chamber views [8]. Although volumetry does not provide information on the function of the left atrium, it nevertheless plays an important role in prognostic assessments [6,9,10]. Measurement of LA strain is mainly used to assess diastolic LV function and is also performed in the two-chamber and four-chamber views. Three components of LA strain can be distinguished: the reservoir function (filling of the LA), the conduit function (opening of the mitral valve and filling of the LV) and the pump function (contraction of the LA). The LA S_R (Left Atrial Stain during Reservoir phase), which reflects the dilatation of the atrium, yields positive values with a reference of 39%; Conduit Strain (LA S_CD) and Left Atrial Contraction Strain (LA S_CT) are given as negative numbers with normal values of −23% or −17%, respectively [11,12]. In particular, LA S_R has been reported to be an important predictor of reduced exercise capacity and therefore an important marker for assessing the prognosis of heart failure, irrespective of its etiology [13,14]. Figure 1 illustrates the longitudinal strain of the left atrium over the cardiac cycle, highlighting the reservoir, conduit and contractile phases, respectively.

Left ventricular ejection fraction (LVEF) is the most widely used parameter in transthoracic echocardiography to assess systolic LV function. Despite some limitations, LVEF is of major importance for the classification of the different phenotypes of heart failure [4]. However, the current literature increasingly points to the potentially higher prognostic value of myocardial strain analysis.

In patients with heart failure, a combination of lifestyle modifications and pharmacological therapies forms the therapeutic basis [15,16]. In addition, cardiac devices, such as implantable cardioverter defibrillators (ICD) and, if indicated, cardiac resynchronization therapy (CRT), can be implemented. For patients with HFrEF and no indication for CRT, cardiac contractility modulation (CCM) is a further treatment option. Based on study results to date, CCM therapy appears to be particularly beneficial in symptomatic patients with an LVEF of 35% or less [17,18].

CCM technology aims to increase the contractile function of the heart by modifying intracellular signaling pathways in cardiomyocytes. It is based on non-excitatory stimulation during the absolute refractory period, which increases calcium influx, prolongs the action potential duration and thus improves the mechanical performance of the myocardium. Clinical and registry-based studies have shown that CCM can enhance systolic function and hemodynamics in chronic heart failure [17,19,20,21,22,23,24,25,26,27,28].

Although several studies have demonstrated an increase in left ventricular function induced by CCM, it is still unclear whether and to what extent CCM might also influence right ventricular and left atrial function, respectively. These questions should be addressed by the current study.

## 2. Methods

We conducted a prospective clinical trial at the Department of Internal Medicine II, Catholic Hospital “St. Johann Nepomuk”, Erfurt, Germany. During the period from 30 September 2021 to 10 September 2024, patients over 18 years of age with reduced left ventricular function and a clinical indication for CCM therapy (QRS complex of less than 130 ms, signs and symptoms of chronic heart failure despite guideline-directed medical therapy) were eligible for inclusion into the study. The trial was approved by the Ethics Committee of the Medical Association of Thuringia and is registered in the German Clinical Trials Register (DRKS00027533). After obtaining written consent, demographic data (age, sex, height, weight), comorbidities and current medications were recorded. Additionally, NT-pro BP levels and eGFR were determined. Standardized echocardiographic examinations were conducted at baseline, 3 and 6 months, respectively, using Vivid E95 system (GE healthcare, Chicago, IL, USA), with a detailed analysis of left and right ventricular function and the determination of LV, RV and LA strain, respectively. Quality of life was evaluated using the Kansas City Cardiomyopathy Questionnaire (KCCQ). Patients were followed every three months, with documentation of CCM therapy status and repeated echocardiographic assessment. Data regarding LV function have been reported previously [29].

During the follow-up period, CCM implantation was performed in 19 patients at a mean time of 59 ± 65 days after baseline. After implantation, CCM therapy was delivered for 6.3 ± 2.3 h per day (standard setting: 1 h “on” followed by 2.25 h “off”; voltage 7.5 V/duration 20.5 ms).

## 3. Statistics

Statistical analysis was performed using SPSS (version 29) (IBM, Armonk, NY, USA). Metric variables are presented as means with standard deviation. For comparisons between two groups, normal distribution was first tested using the Shapiro–Wilk test. If a normal distribution was found, the student’s t-Test was used; otherwise, the Mann–Whitney U test was used. Data labelled as “no active CCM” refer to measurements taken before CCM-implantation, whereas “CCM active” refers to measurements under active CCM therapy.

## 4. Results

Between 30 December 2021 and 10 September 2024, 22 patients were included in the study. The baseline parameters of these patients are provided in Table 1.

### Effects of CCM Therapy

**Figure 2:**
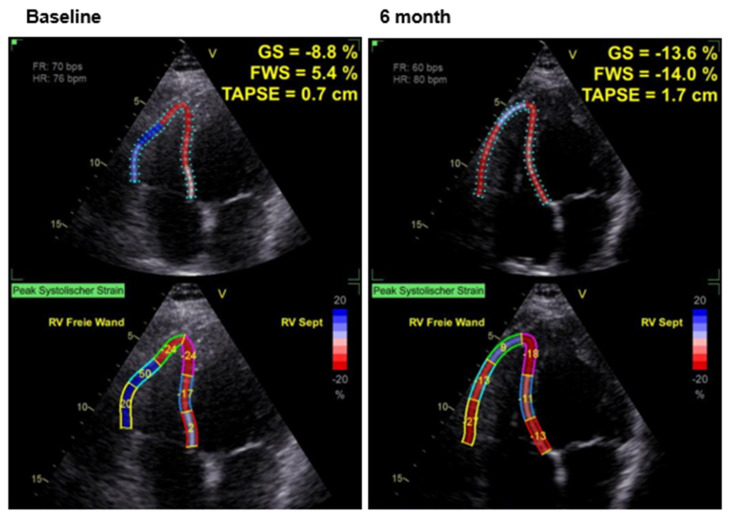
Example of right ventricular strain in a patient at baseline and after 6 months of CCM therapy.

**Figure 3:**
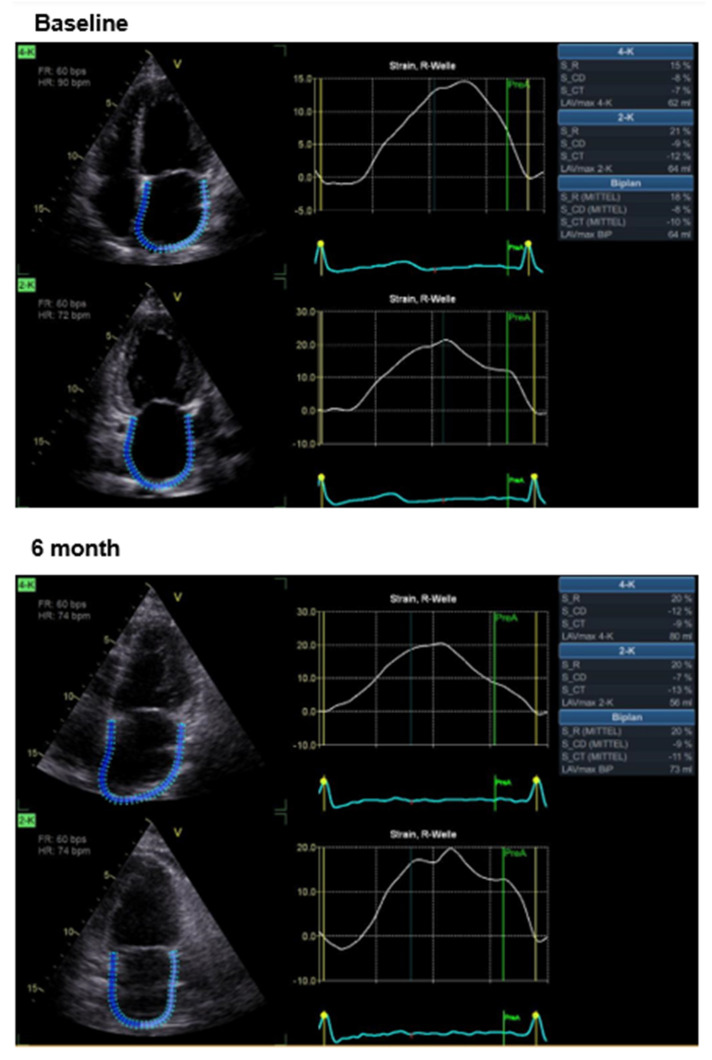
Example of left atrial strain in a patient at baseline and after 6 months of CCM therapy.

The effects of CCM therapy on RV and LA strain as well as volumetric and functional parameters of the right ventricle are shown in Table 2; the effects on parameters of LV function have already been published previously [29]. Under active CCM therapy, improved values for RV GS, FWS, LA S _R and LA S_CT are found. Figure 2 and Figure 3 illustrate examples of the impact of cardiac contractility modulation (CCM) therapy on right ventricular strain and on left atrial strain, respectively.

**Figure 4:**
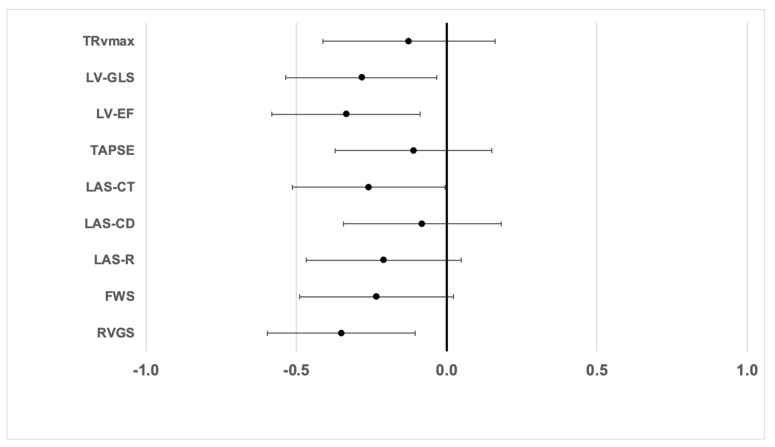
Influence of CCM therapy on echocardiographic parameters of LV, LA and RV function, respectively. The graph shows the standardized regression coefficients (linear regression model) with 95% confidence intervals to allow a comparison of the effect sizes.

In order to compare the effect size of CCM therapy on the different echocardiographic parameters, a linear regression model was used. Figure 4 shows the standardized regression coefficients with 95% confidence intervals from this analysis, showing that CCM leads to comparable improvements of both LV (LVEF, GLS) and RV function (RV GS). Additionally, in this model, active CCM therapy was also associated with a significant increase in LA contraction strain (LAS CT).

We have shown previously that CCM therapy leads to an improvement of quality of life in patients with HFrEF, as seen from the increase of the KCCQ score [29]. To explore which echocardiographic parameters are linked to quality of life, we again used a linear regression model, with KCCQ as the outcome variable. Surprisingly, it turns out that parameters of LA strain seem to be especially linked to the KCCQ score, whereas neither RV strain nor LV GLS were significantly associated to quality of life in our analysis (Table 3). Furthermore, even when both age and NTproBNP levels were introduced into the regression model as confounding variables, LAS-CT continued to show a clear association with KCCQ (standardized beta: −0.28; *p* < 0.05).

## 5. Discussion

Several studies have proven that the implementation of cardiac contractility modulation leads to an improvement of both LV-EF and LV global longitudinal strain in patients with HFrEF, which is paralleled by an improved quality of life and prognosis [27,29,30,31]. The aim of the analysis presented here was to investigate whether CCM would also have an impact on the strain of the right ventricle or the left atrium, respectively. Our data now show for the first time that both RV and LA strains are indeed increased under active CCM therapy. This is of particular interest as both RV and LA strains have been reported to be closely associated with the prognosis of patients with HFrEF and HFpEF [13,14,32].

It is well established that cardiac resynchronization therapy (CRT) leads to a better prognosis in patients with HFrEF. Besides its well-known effects on LV function, a recent meta-analysis of 30 studies showed that CRT also significantly influences right ventricular function. In particular, right ventricular fractional area change, tricuspid annular plane systolic excursion (TAPSE), and systolic pulmonary artery pressure were all positively affected by CRT [30], and it can be assumed that these effects also contribute to the clinical benefit of this therapy. In parallel, our data now show that similar findings regarding RV function also exist in heart failure patients under CCM therapy.

There are numerous studies that have demonstrated the prognostic impact of several echocardiographic parameters, including right ventricular global strain and left atrial strain, in patients with heart failure. Likewise, one study involving 1089 patients showed that both TAPSE and RV strain are independent predictors of two-year mortality in patients with tricuspid regurgitation [31], and improvements in these parameters are closely associated with enhanced survival rates [32]. However, so far there are only very few studies that describe an association between quality of life and distinct echocardiographic parameters in patients with heart failure. A study in healthy older adults could not detect a correlation of either RV strain or TAPSE with the KCCQ score [33]. Interestingly, left atrial conduit strain has been reported to be significantly associated with improved KCCQ scores following edge-to-edge repair of mitral regurgitation [34]. In line with this, our data indicate that among all tested parameters, improvements in LA strain show the strongest correlation with quality of life, as measured by KCCQ.

LA strain is increasingly recognized as a sensitive marker for diastolic function and the severity of heart failure. Specifically, a Left Atrial Stain during Reservoir phase below 18% is associated with an increased pulmonary wedge pressure (PCWP), making it particularly useful for the evaluation of diastolic function [35]. Interestingly, in animal studies in rabbits with chronic heart failure (CHF), CCM led to the significant downregulation of TGF-β1 and Smad3, which was associated with reduced collagen deposition and fibrosis [36], which in turn could be a molecular explanation for the improvement of diastolic function. Furthermore, cardiac contractility modulation (CCM) has been reported to affect the myocardial protein titin by increasing its phosphorylation. This modification can improve myocardial relaxation and positively affect diastolic function as well, which is considered an additional mechanism of action of CCM in heart failure [37]. Furthermore, CCM has been shown to promote the interaction of titin with protective proteins such as α-crystallin B chain, which can additionally support the function and stability of titin [38].

In a study of 106 patients with heart failure (HFrEF), reduced atrial longitudinal strain was associated with a significantly worse prognosis for cardiovascular death or hospitalization [39]. Therapeutic measures that lead to an improvement of LA strain could therefore potentially both be of prognostic benefit and increase the quality of life in patients with heart failure. In our study, both LA and RV strains were improved by approximately 4% by CCM therapy (Table 2). To the best of our knowledge, no previous reports have quantitatively related changes in LA or RV strain to clinical outcomes. Therefore, it is rather difficult to judge whether the observed magnitude of improvement seen in our study will finally translate into prognostic benefits. On the other hand, CCM is an established therapy leading to better clinical outcomes in chronic heart failure including very challenging scenarios [17,40], so it can be assumed that the improvement of LA and RV strains might also contribute to these effects.

### Limitations

One must take into consideration that the study includes only 22 patients, which somewhat limits generalizability and statistical power. Due to the limited number of patients and the study protocol, our study was not designed to provide statistical information on the effects of CCM on the incidence of clinical endpoints, such as hospitalization for heart failure or mortality. In addition, the results may have been influenced by placebo effects due to the open-label design of the study. Furthermore, the average interval between baseline assessment and CCM implantation was 59 ± 65 days, which could potentially have had an influence on the observed strain parameters.

## Figures and Tables

**Table 1:** Demographic parameters: estimated glomerular filtration rate (GFR), NT-pro BP, and medical history at baseline.

Parameter	Mean (All Patients; N = 22)	Ischemic Cardiomyopathy [11]	Non-Ischemic Cardiomyopathy [11]
Male sex	16 (73%)	9 (81.8%)	8 (63.6%)
Age [years]	69.6 ± 6.4	69.0 ± 7.7	70.3 ± 5.1
Hight [cm]	170.6 ± 10.0	174.1 ± 9.1	167.1 ± 10.1
Weight [kg]	95.5 ± 21.9	100.0 ± 23.2	91.0 ± 20.5
GFR [ml/min*1.73 m^2^]	62.4 ± 17.5	60.3 ± 13.0	64.4 ± 21.5
NT-pro BP [pg/mL]	2669.2 ± 3716.8	1528.5 ± 1809.0	3810.0 ± 4781.2
NYHA stage			
NYHA I	0.0 (0%)	0 (0.0%)	0 (0.0%)
NYHA II	4 (18.18%)	4 (36.4%)	0 (0.0%)
NYHA III	12 (54.6%)	6 (54.5%)	6 (54.5%)
NYHA IV	6 (27.3%)	1 (9.1%)	5 (45.5%)
Hypertension	18 (82.8%)	9 (81.8%)	9 (81.8%)
Diabetes mellitus	14 (64.6%)	9 (81.8%)	5 (45.5%)
Coronary heart disease	11 (50.0%)	11 (100.0%)	0 (0.0%)
History of PCI	11 (50.0%)	11 (100.0%)	0 (0.0%)
History of CABG	1 (4.6%)	1 (9.1%)	0 (0.0%)
History of heart value surgery	6 (27.3%)	3 (27.3%)	3 (27.3%)
Antiplatelet therapy (APT)			
Single APT	9 (40.9%)	6 (54.5%)	3 (27.3%)
Dual APT	2 (9.1%)	1 (9.1%)	1 (9.1%)
Non	11 (50.0%)	4 (36.4%)	7 (63.6%)
(D)OAC	11 (50.0%)	6 (54.5%)	5 (45.5%)
ß-blockers	19 (86.4%)	10 (90.9%)	9 (81.8%)
ACE inhibitors	3 (13.6%)	2 (18.2%)	1 (9.1%)
ARB	1 (4.6%)	1 (9.1%)	0 (0.0%)
Sacubitril/Valsartan	18 (81.8%)	8 (72.7%)	10 (90.9%)
MRA	15 (68.2%)	6 (54.5%)	9 (81.8%)
SGLT2 inhibitors	14 (64.6%	7 (63.6%)	7 (63.6%)
Diuretic KCCQ	19 (86.4%) 31.3 ± 16.8	10 (90.9%) 37.7 ± 19.0	9 (81.8%) 24.9 ± 11.8

**Table 2:** Parameters of transthoracic echocardiography depending on the delivered therapy (CCM active or no active CCM).

Parameter	CCM Active (N = 22)	No Active CCM (N = 39)	*p*-Value
RV GS [%]	−13.7 ± 4.5	−10.1 ± 5.0	<0.05
FWS [%]	−14.6 ± 7.3	−10.3 ± 10.2	<0.05
LA S_R [%]	19.7 ± 1.0	15.3 ± 10.2	<0.05
LA S_CD [%]	−9.0 ± 5.0	−8.1 ± 5.4	n.s.
LA S_CT [%]	−11.5 ± 7.0	−7.1 ± 8.5	<0.05
TAPSE [mm]	18.8 ± 5.1	17.6 ± 5.6	n.s.
Trvmax [m/s]	2.6± 0.4	2.9 ± 1.3	n.s.
RVDd [mm]	39.2 ± 16.9	38.8± 7.5	n.s.
RVDs [mm]	30.2 ± 17.5	30.3 ± 7.0	n.s.

RV GS: global right ventricular strain; FWS: right ventricular free wall strain; LA S_R: Left Atrial Stain during Reservoir phase; LA S_CD: Left Atrial Conduit Strain; LA S_CT: Left Atrial Contraction Strain; TAPSE: Tricuspid Annular Plane Systolic Excursion; Trvmax: maximum velocity of tricuspid regurgitation; RVDd: diameter of the right ventricle in diastole; RVDs: diameter of the right ventricle in systole. n.s.: not significant

**Table 3:** Influence of echocardiographic parameters on quality of life (KCCQ score) in patients with HFrEF (linear regression analysis; univariate analysis).

Variable	Standardized Beta	*p*-Value
Age	−0.112	0.39
LVEF	0.163	0.210
LV GLS	0.055	0.673
TRVmax	−0.173	0.23
RV GS	−0.147	0.261
FWS	−0.070	0.597
LAS_R	0.270	0.037
LAS_CD	−0.081	0.536
LAS_CT	−0.321	0.012

## Data Availability

Data can be obtained from the authors on reasonable request.
